# N-terminal deletion of Swi3 created by the deletion of a dubious ORF *YJL175W* mitigates protein burden effect in *S. cerevisiae*

**DOI:** 10.1038/s41598-020-66307-z

**Published:** 2020-06-11

**Authors:** Nozomu Saeki, Yuichi Eguchi, Reiko Kintaka, Koji Makanae, Yuichi Shichino, Shintaro Iwasaki, Manabu Kanno, Nobutada Kimura, Hisao Moriya

**Affiliations:** 1 Graduate School of Environmental and Life Science, Okayama University, Okayama, Japan; 2 Center for Mechanisms of Evolution, School of Life Sciences, Arizona State University, Arizona, USA; 3 Donnelly Center for Cellular and Biomolecular Research, Department of Medical Genetics, University of Toronto, Toronto, Canada; 4 RNA Systems Biochemistry Laboratory, RIKEN Cluster for Pioneering Research, Saitama, Japan; 5 Department of Computational Biology and Medical Sciences, Graduate School of Frontier Sciences, The University of Tokyo, Chiba, Japan; 6 Bioproduction Research Institute, National Institute of Advanced Industrial Science and Technology, Ibaraki, Japan; 7 Research Core for Interdisciplinary Sciences, Okayama University, Okayama, Japan

**Keywords:** Cell growth, Gene expression, Gene regulation

## Abstract

Extreme overproduction of gratuitous proteins can overload cellular protein production resources, leading to growth defects, a phenomenon known as the protein burden/cost effect. Genetic screening in the budding yeast *Saccharomyces cerevisiae* has isolated several dubious ORFs whose deletions mitigated the protein burden effect, but individual characterization thereof has yet to be delineated. We found that deletion of the *YJL175W* ORF yielded an N-terminal deletion of Swi3, a subunit of the SWI/SNF chromatin remodeling complex, and partial loss of function of Swi3. The deletion mutant showed a reduction in transcription of genes encoding highly expressed, secreted proteins and an overall reduction in translation. Mutations in the chromatin remodeling complex could thus mitigate the protein burden effect, likely by reallocating residual cellular resources used to overproduce proteins. This cellular state might also be related to cancer cells, as they frequently harbor mutations in the SWI/SNF complex.

## Introduction

Expression levels of intracellular proteins are tightly controlled to maintain organism capacity for proliferation and survival, and an excess of proteins can cause cellular dysfunction^1–3^. Potentially, any harmless protein inhibits cell growth when it is extremely overproduced, because it depletes cellular protein production resources. This phenomenon is known as the protein burden/cost effect^3–6^. The protein burden effect was initially observed as growth defects of bacterial cells overexpressing gratuitous proteins^7^, and later analyzed in yeast as well^2,4,6,8^. The protein burden effect is triggered by the cost of gene expression upon overexpression of gratuitous proteins; the overexpression overloads cellular transcription and translation resources^4,9^. Because the protein burden effect is triggered by the massive overexpression of unneeded proteins, cancer cells, where an increase in the chromosome numbers is common^10,11^, should be under the condition of the protein burden effect. While the protein burden effect initially appears to be a simple phenomenon, little is known about the physiological conditions and cellular responses triggered by the protein burden effect.

Extreme overexpression of fluorescent proteins such as GFP and RFP are thought to trigger this effect^4,6,8,12^. To clarify the physiology of cells suffering from protein burden effect, we recently conducted genetic profiling^13^. Upon isolating a series of deletion and temperature-sensitive mutants harboring genetic interactions affecting the overproduction of GFP (GFP-op) in the budding yeast *Saccharomyces cerevisiae*, we found that the deletion of certain uncharacterized ORFs mitigated growth defects triggered by GFP-op. However, the molecular details underpinning alleviation of the protein burden effect in these mutants remained unclear.

The SWI/SNF complex is a chromatin remodeling complex that remodels nucleosomes and changes chromatin structure by using the hydrolysis energy of ATP^14^. The SWI/SNF complex is evolutionarily conserved in eukaryotes^15^. In humans, the complex is also known as BAF or PBAF complex^15^. The SWI/SNF complex is composed of 12 subunits in budding yeast and 11–15 subunits in human^16,17^. The SWI/SNF complex has Swi2/Snf2 as the ATPase component, Swi3 and Snf5 as the core subunits, and several other accessory subunits. 20% of all human cancers have mutations in the SWI/SNF complex subunits^18^. For instance, 98% of Rhabdoid tumors and 20–40% of Familial schwannomatosis have a homozygous deletion or truncating mutation in SNF5^19^. However, the underlying mechanism relating to the mutations in the SWI/SNF complex and cancer physiology is still unclear.

In this study, we characterized the yeast deletion mutants in which the growth defects triggered by GFP-op are mitigated, and revealed that one of the deletion mutants unexpectedly created an N-terminal deletion of *SWI3*, a component of the SWI/SNF complex, and a reduction in transcription levels of certain genes. We thereby suggest that transcriptional alterations may free up ribosomes to accept ectopically expressed mRNA for translation and mitigate the protein burden effect.

## Results

### Deletion of *YJL175W* ORF mitigates GFP-op-triggered growth defects

**Figure 1:**
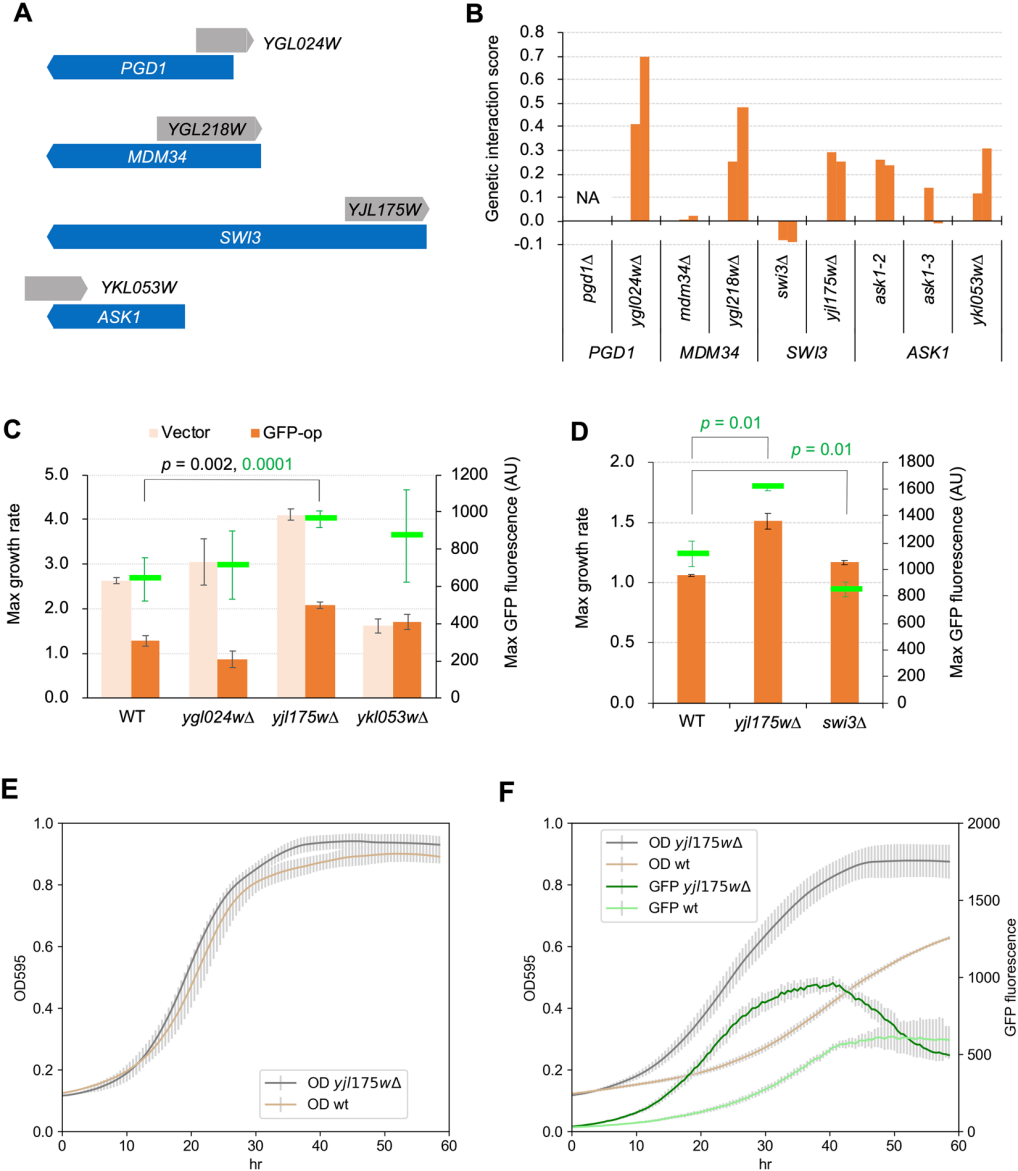
Deletion of *YJL175W* mitigates GFP-op-triggered growth defects. (**A**) Dubious ORFs of whose deletions showed positive interactions with GFP-op overlapping other ORFs. Gray and blue arrows show dubious ORFs, and the verified ORFs overlapped with them, respectively. (**B**) Genetic interaction scores of indicated mutants with GFP-op. For each mutant, the scores from two independent experiments are shown. Data were obtained from Kintaka *et al*.^13^. Genetic interaction score was calculated from colony size differences among control strains. NA: not analyzed. (**C**,**D**) Max growth rates (orange bars) and max GFP fluorescence levels (green boxes) of wild type (WT) and indicated mutants grown in the synthetic medium (−Leu/Ura). (**C**) The max growth rate and max GFP fluorescence *p*-values. (**D**) The max GFP fluorescence *p*-values. (**E**) Growth curves of WT and the *YJL175W* deletion mutant with empty vector in synthetic medium (−Leu/Ura). (**F**) Growth curves and GFP fluorescence of WT and the *YJL175W* deletion mutant upon GFP-op in synthetic medium (−Leu/Ura).

Overproduction of GFP (yEGFP3) under the control of strong *TDH3* promoter from a multicopy plasmid pTOW40836 causes growth defects, probably due to the protein burden effect^6^ (Fig. 1C). We recently performed a systematic screening of deletion mutants and temperature-sensitive mutants in which GFP-op triggered growth defects were aggravated or mitigated^13^. We performed the synthetic genetic array analysis^20^ to obtain genetic interaction scores between GFP-op and the mutants. The genetic interaction score indicates how much the growth of GFP-op in a mutant differs from what is expected from each of the growth of GFP-op in the wild type and the growth of the vector control in the mutant. If the score is negative (namely the mutant negatively interacts with GFP-op), the growth defect triggered by GFP-op is aggravated. While if the score is positive (namely the mutant positively interacts with GFP-op), the growth defect is mitigated.

The screening isolated 100 mutants positively interact with GFP-op^13^, and the mutants contained the deletions of four dubious ORFs; *YGL024W*, *YGL218W*, *YJL175W*, and *YKL053W*. These ORFs overlap with and are located on opposite strands from the verified genes *PGD1*, *MDM34*, *SWI3*, and *ASK1* and are thus unlikely to encode functional proteins (*Saccharomyces* Genome Database, Fig. 1A). Figure 1B shows the genetic interaction scores between GFP-op and the indicated mutants. All *ykl053wΔ* and TS mutants of *ASK1* (*ask1-2* and *ask1-3*) demonstrated positive interactions, suggesting that *ykl053wΔ* disrupts the function of the *ASK1* gene. Conversely, although *ygl218wΔ* and *yjl175wΔ* yielded positive interactions, *mdm34Δ* and *swi3Δ* did not. This suggests that the deletions of *YGL218W* and *YJL175W* result in different consequences due to loss of function of *MDM34* and *SWI3*.

We then analyzed growth rates and GFP levels in liquid media to confirm the positive interactions between GFP-op and deletion mutants of three dubious ORFs. Among the three deletion mutants, only *yjl175wΔ* showed a significantly higher growth rate (*p* = 0.002; Fig. 1C); *yjl175wΔ* also presented with higher GFP levels than wild type (WT) cells (*p* = 0.0001; Fig. 1C). This phenotype was not observed in *swi3Δ* cells wherein GFP levels were lower than those in WT cells (*p* = 0.01; Fig. 1D), indicating that *yjl175wΔ* does not cause loss of function by *SWI3*. Figure 1E,F show the growth curves and GFP expression levels of *yjl175wΔ* and WT cells, and the dramatic decrease in growth rate triggered by GFP-op in WT was not observed in these cells. We thus focused on *YJL175W*-*SWI3* for subsequent analysis.

### Deletion of *YJL175W* leads to partial loss of function of Swi3

**Figure 2:**
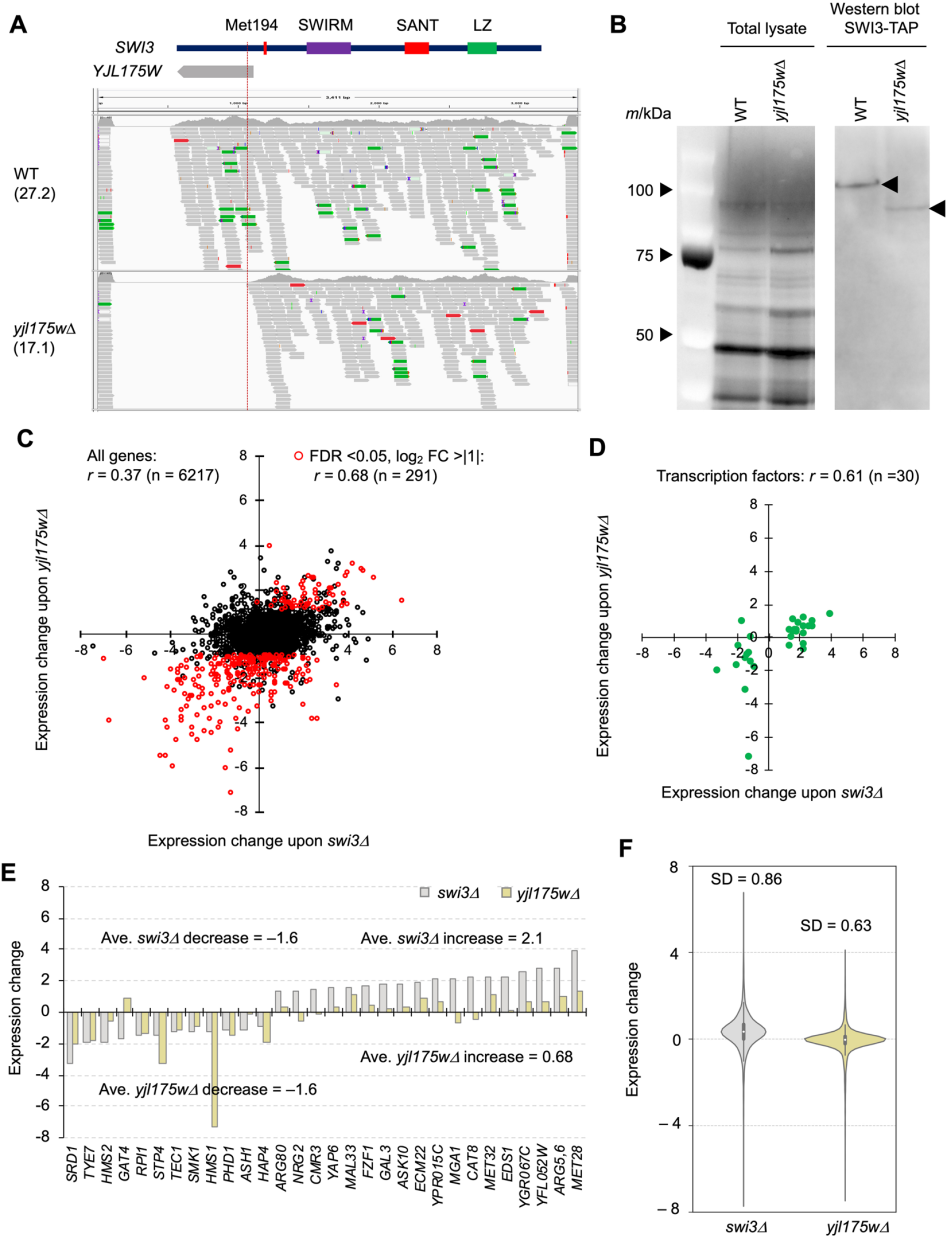
Deletion of *YJL175W* creates partial loss of function of Swi3. (**A**) RNA-seq reads mapped to the *YJL175W*-*SWI3* locus in the wild type (WT) and *yjl175w∆* transcripts displayed using IGV (2.4.9). Corresponding locations of Swi3 domains and *YJL175W* are also shown. Dotted line represents the predicted transcript end of truncated *SWI3* in *yjl175w∆*. Expression levels of the *SWI3* transcripts (TPM) in each cell are also shown. (**B**) Truncated form of Swi3 expressed in *yjl175w∆* cells. The TAP-tag was fused to the C-terminus of *SWI3* in WT and *yjl175w∆*, and Swi3-TAP was then detected by Western blot. Total cellular protein content is also shown. The full-length gel and blot images are shown in Fig. S5. (**C**) Relationship between expression changes of transcripts upon *SWI3* and *YJL175W* deletion. Red dots show transcripts with a false discovery rate (FDR) < 0.05 and log_2_ FC > |1|. (**D,E**) Expression changes of transcripts of transcription factors under *swi3∆* and *yjl175w∆*. Only transcription factors known to be affected by the *SWI3* deletion^16^ are shown. In E, average log_2_ expression changes of increased and decreased genes under *swi3∆* and *yjl175w∆* are shown. (**F**) Distributions of expression changes of transcripts in *swi3*∆ and *yjl175w∆*. The sample number is represented by *n* and the Pearson correlation coefficiency by *r*. Expression change is shown as log_2_ FC over WT.

We then performed transcriptome (RNA-seq) analyses to elucidate the consequences of *YJL175W* deletion. We first analyzed the transcripts expressed at the *YJL175W*-*SWI3* locus (Fig. 2A). Although the deletion of *YJL175W* removes the 5′ region of *SWI3* (Figs. S1 and S2), partial *SWI3* transcripts were still expressed, with an estimated expression level at about 63% of WT. The end of the transcript (dotted line, Fig. 2A) suggests that the deletion of *YJL175W* produced a truncated Swi3 lacking its N-terminal 193 amino acid (Fig, S2). Swi3 is a subunit of the SWI/SNF chromatin remodeling complex^21^, and known functional domains of Swi3 are located at the C-terminus^22,23^; the truncated Swi3 contains all three domains, SWIRM, SANT, and LZ (Fig. 2A), suggesting that a truncated Swi3 retains some function. Western blot analysis validated the expression of truncated Swi3 in *yjl175wΔ* cells (Fig. 2B).

To further assess the effects of *YJL175W* deletion, we next compared the transcriptional profiles of *swi3Δ*^16^ and *yjl175Δ* (Supplementary data set). Complete transcriptional changes associated with *SWI3* and *YJL175W* deletions were weakly correlated (*r* = 0.37) (dots, Fig. 2C), a correlation that increased (*r* = 0.68) when transcripts with expressions significantly changed by *yjl175wΔ* were compared (red dots, Fig. 2C). The previous study demonstrated that the expression levels of mRNAs encoding transcription factors were significantly altered in *swi3∆* cells^16^, and indeed, the *YJL175W* deletion presented with expression changes in transcription factors similar to those associated with *SWI3* deletion (*r* = 0.61; Fig. 2D,E). These results suggest that the *YJL175W* deletion resulted in a similar transcriptional change as the deletion of *SWI3*. However, the transcriptional change range was much wider in *swi3Δ* cells (standard deviation (SD) = 0.86) than that in *yjl175wΔ* cells (SD = 0.63; Fig. 2F), indicating that the deletion of *YJL175W* resulted in less pronounced transcriptional changes than the deletion of *SWI3*. This difference in transcriptional changes was also observed for the expression of transcription factors; although changes in the transcription factors were equally distributed in *swi3∆* cells, the transcriptional decrease was far greater than the increase in *yjl175∆* cells (Fig. 2E).

We thereby concluded that the *YJL175W* deletion caused partial loss of function of *SWI3*, particularly related to the activation of transcription of Swi3 targets, as the underlying cause of the difference between the phenotypes of *swi3∆* and *yjl175w∆* shown in Fig. 1.

### Transcriptional consequences of N-terminal deletion of Swi3

**Figure 3:**
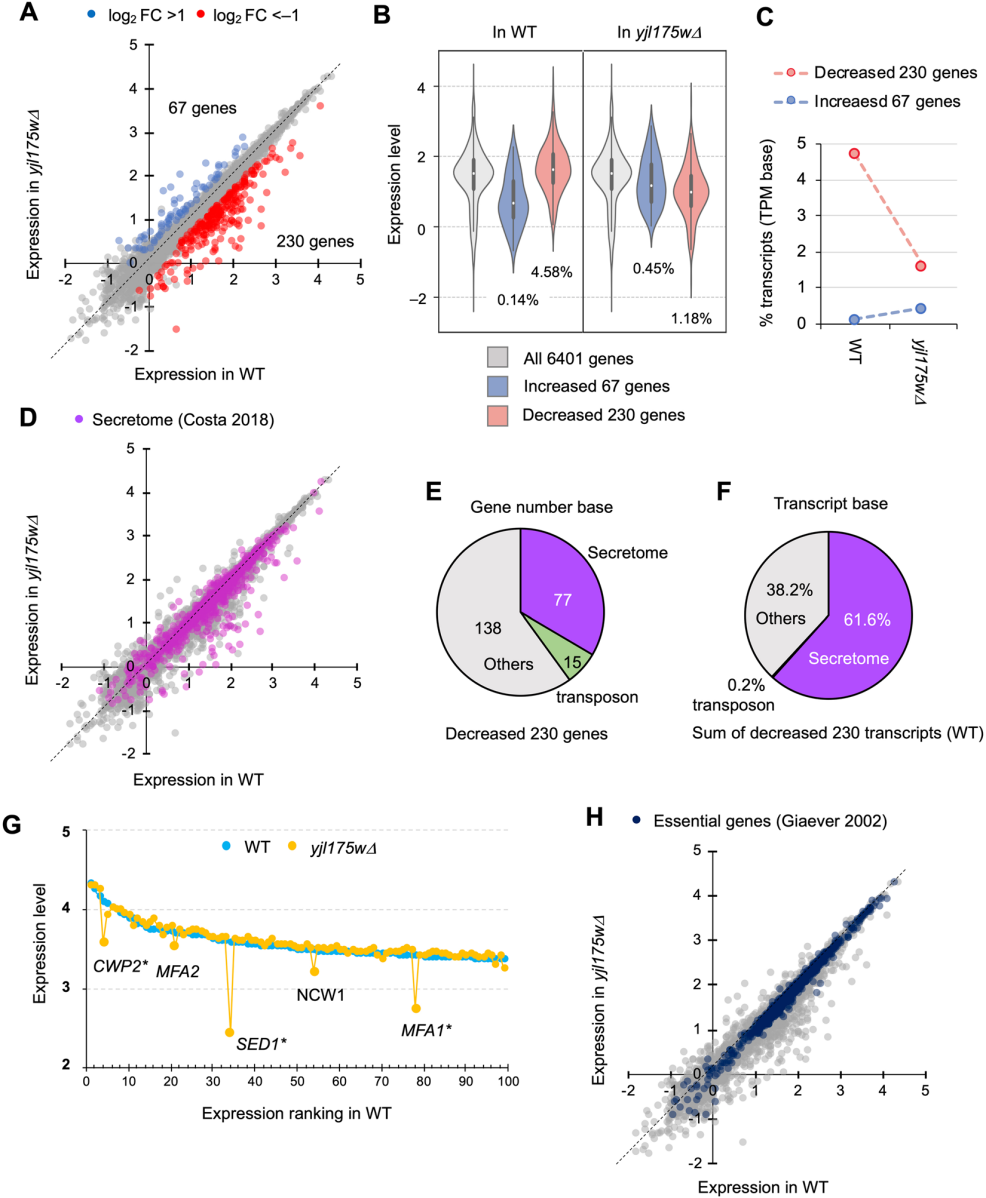
Transcriptional consequences of deletion of *YJL175W*. (**A**) Comparison of transcript levels between WT and *yjl175w∆*. Blue and red dots indicate significantly increased transcripts (FDR < 0.05 and log_2_ FC > 1) and decreased transcripts (FDR < 0.05 and log_2_ FC < − 1), respectively. Numbers of increased and decreased genes are also shown. (**B**) Distributions of all, 67 increased, and 230 decreased transcripts upon *YJL175W* deletion; their proportions in WT and in *yjl175w∆* transcripts are also shown as the percentage. The percentage was calculated as follows: (the sum of TPMs of 67 increased or 230 decreased transcripts)/(the sum of TPMs of all 6401 genes) × 100. (**C**) Alteration of transcripts upon deletion of *YJL175W*. The transcript percentage was calculated as in (**B**). (**D**) Expression levels of transcripts coding for secreted proteins (secretome) in WT and *yjl175w∆*. Purple dots show transcripts of secretome proteins. The secretome protein list (1088 proteins) was obtained from Costa *et al*.^28^. (**E**,**F**) Proportions of the numbers of genes (**E**) and sums of transcripts (**F**) of secretome proteins in the 230 genes significantly decreased upon *YJL175W* deletion. Transcript sum is calculated by the sum of the TPMs of contained transcripts. (**G**) Expression levels of the top 100 highly expressed genes in WT and *yjl175w∆*. Five representative genes with expression levels significantly reduced upon *YJL175W* deletion are shown. Genes with asterisks showed a greater than twofold decrease upon *YJL175W* deletion. The adjusted *p*-values of the expression changes of indicated genes were <2.1E-30 (Table S3) (**H**). Expression levels of transcripts of essential genes in WT and *yjl175w∆*. Dark blue dots represent transcripts of essential genes. Essential gene list (1274 genes) was obtained from Giaever *et al*.^29^. Expression change is shown as log_10_ TPM.

We then analyzed the transcriptional profile of *yjl175wΔ* in more detail to further delineate the transcriptional consequences of the N-terminal deletion of Swi3 (*swi3ΔN*) created by the deletion of *YJL175W*. Figure 3A shows variations in transcripts between WT and *yjl175w∆* cells. The number of decreased genes (230) was about 3.4 times higher than the number of increased genes (67). Overall alterations of expression levels of these transcripts upon *yjl175w∆* are shown in Fig. 3B. The decreased 230 genes constituted 4.58% of the total transcripts in WT cells and were decreased to 1.18% in *yjl175w∆*. The increased 67 transcripts constituted only 0.14% of total WT transcripts and were increased to only 0.45% in *yjl175w∆* (Fig. 3B). This asymmetrical distribution, showing more decreased transcripts than increased ones (Fig. 3C), suggests an overall reduction of transcription in *yjl175w∆* cells compared with that in WT cells. Of note, however, these ratios do not necessarily reflect intracellular mRNA ratios, as the number of each transcript is normalized to the number of total transcripts.

We then performed gene ontology (GO) enrichment analysis to reveal processes and genes affected in th*e yjl175w∆* cells (Tables 1, 2 and S1). The set of 67 increased genes was enriched with GOs related to methionine biosynthetic processes, likely associated with an increase in the transcription factors *MET28* and *MET32* (Fig. 2E)^24,25^. The set of 230 decreased genes was enriched with GOs related to transposon activity, which is likely associated with a decrease in the transcription factors *TYE7* and *TEC1* (Fig. 2E)^26,27^. Decreased genes were significantly enriched with GO genes for secreted proteins^28^ (Table 2, GO0071944: cell periphery); in fact, 77 out of the 230 decreased genes encoded secretory proteins (Fig. 3D,E), a level that was significantly higher than expected (*p* = 1.62E–26). Transcripts of secreted proteins accounted for more than 60% of the decreased transcripts, whereas transposon-related transcripts accounted for only 0.2% (Fig. 3F), suggesting that those secreted proteins were highly expressed. Figure 3G shows the expression levels of the top 100 highly expressed genes in WT. Specific reductions in *yjl175w∆* transcripts encoding secretory proteins, such as *CWP2*, *MFA2*, *SED1*, *NCW1*, and *MFA1*, were observed, and no transcript increased more than twofold. The decreased secretory genes were not essential for viability^29^, and the *yjl175w∆* cells did not present with reductions in growth under normal growth conditions (Fig. 1E). We thereby speculated that the *YJL175W* deletion might only affect the transcription of non-essential genes. In fact, essential genes made up just 5% (15 genes) of the 297 genes with expression levels altered by more than twofold (Fig. 3H), which was far fewer than expected (*p* = 1.82E–15).

**Table 1:** GO analysis of 67 genes increased upon deletion of *YJL175W*.

GO term	*p*-value	GO ID	Matches
**Biological process**			
cysteine biosynthetic process^*^	1.94E-09	0019344	7
sulfur amino acid metabolic process^*^	7.71E-09	0000096	10
methionine biosynthetic process^*^	1.08E-08	0009086	9
**Molecular function**			
sulfur compound transmembrane transporter activity^*^	0.00359535	1901682	4
anion transmembrane transporter activity^*^	0.0069415	0008509	7
sulfite reductase (NADPH) activity^*^	0.00835406	0004783	2
**Cellular component**			
sulfite reductase complex (NADPH)^*^	0.00499521	0009337	2

Three GOs with highest *p*-values are shown. ^*^GO related to methionine-biosynthesis. A full list is provided in Table S1.

**Table 2:** GO analysis of 230 genes decreased upon deletion of *YJL175W*.

GO term	*p*-value	GO ID	Matches
**Biological process**			
DNA integration^*^	3.01E-07	0015074	14
generation of precursor metabolites and energy	8.06E-06	0006091	25
transposition, RNA-mediated^*^	3.72E-05	0032197	17
**Molecular function**			
aspartic-type endopeptidase activity^*^	2.31E-08	0004190	15
aspartic-type peptidase activity^*^	2.31E-08	0070001	15
RNA-DNA hybrid ribonuclease activity^*^	6.75E-08	0004523	14
**Cellular component**			
cell periphery^#^	6.43E-11	0071944	66
fungal-type cell wall^#^	5.56E-09	0009277	23
cell wall^#^	1.38E-08	0005618	23

Three GOs with highest *p*-values are shown. ^*^GO related to transposon activity. ^#^GO containing genes encoding secreted proteins. A full list is provided in Table S1.

We thus concluded that the *swi3ΔN* mutant generated by the deletion of *YJL175W* caused a selective transcriptional reduction of genes encoding highly expressed, non-essential secreted proteins.

### N-terminal deletion of Swi3 leads to change in translation status

**Figure 4:**
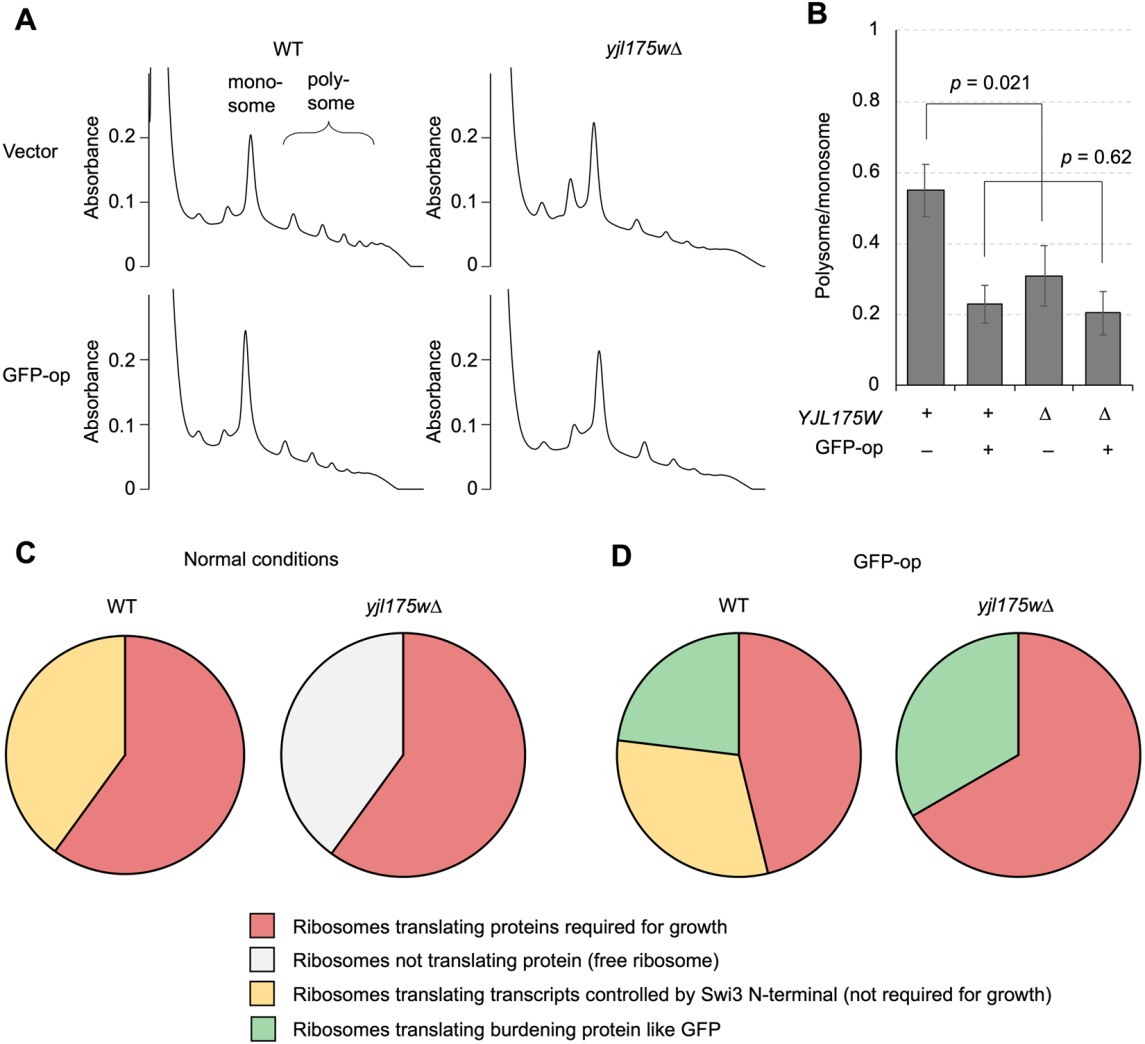
Translational consequence upon deletion of *YJL175W*. (**A**) Polysome profiling of WT and *yjl175w∆* cells under normal (vector) and GFP-op conditions. Predicted monosome and polysome peaks are shown. (**B**) Ratios of polysome to monosome in indicated conditions. (**C**,**D**) Conceptual pie chart models interpreting how translational changes observed in *yjl175w∆* mitigated growth defects upon GFP-op. These charts show the hypothetical allocations of ribosomes in indicated conditions, but their percentages are not based on the real data. See the main text for a detailed explanation of these charts.

Given that *swi3ΔN* (*yjl175w∆*) was shown to mitigate the protein burden effect, we speculated that translation status in the mutant cells affected the ectopic expression of proteins therein. We thus analyzed the polysome profiles of WT and *yjl175w∆* (Fig. 4A) by calculating polysome/monosome ratios (Fig. 4B) to represent translation efficacy in cells. In the vector control, the polysome/monosome ratio was significantly lower in *yjl175w∆* than that in WT; that is, the number of translating ribosomes was lower in *yjl175w∆*. This result suggests that the reduction in transcription of some genes in *yjl175w∆* might lead to a reduction in overall translation, which might free up ribosomes used to produce foreign proteins. Interestingly, the difference in polysome/monosome ratio between WT and *yjl175w∆* cells did not occur under GFP-op conditions (Fig. 4B), suggesting that under such conditions, the same number of ribosomes is engaged in translation in *yjl175w∆* as that in WT, leading to the higher GFP levels observed in the mutant.

## Discussion

In this study, we showed that the deletion of *YJL175W* unexpectedly led to a partial loss of function of Swi3 (*swi3∆N*) and mitigated growth defects triggered by GFP-op, a condition implicated in the protein burden effect. We believe that *YJL175W* is a misannotated ORF because there is no homologous protein even in closely-rerated *Saccharomyces* species (data not shown), and no transcript corresponding to the *YJL175W* locus was detected (Fig. S3). Therefore, the phenotype of the *YJL175W* deletion should be solely created by the truncation of Swi3 accidentally created by the *YJL175W* deletion. A substantial portion of transcription was reduced upon *swi3∆N* via the selective reduction of transcripts encoding highly expressed, secreted proteins (Fig. 3). This transcriptional reduction also led to a reduction in total protein translation (Fig. 4A,B). Cellular conditions created by the *YJL175W* deletion were speculated in accordance with these results. Figure 4C,D show conceptual pie chart models explaining such conditions. In normal conditions, ribosomes are used to translate both proteins that are and are not required for growth (Fig. 4C, WT). In *yjl175w∆* cells, the number of transcripts encoding proteins not required for growth was lower, and consequently, ribosomes tasked with translating those transcripts were freed up to perform other translations (Fig. 4C, *yjl175w∆*). In GFP-op, ribosomes are used to translate GFP, leading to a reduction in the number of ribosomes translating proteins required for growth followed by growth defects (Fig. 4D, WT). Free ribosomes created by *yjl175w∆* reinstates extra ribosomes for GFP translation, avoiding a reduction in the translation of proteins required for growth (Fig. 4D, *yjl175w∆*) and mitigating growth defects.

Although *yjl175w∆* cells have an increased capacity to produce exogenic proteins, this phenomenon is associated with an apparent trade-off. These cells are sensitive to stressors, such as high temperature, alkaline pH, and many chemicals (*Saccharomyces* Genome Database), perhaps due to a transcriptional reduction of stress tolerance-related genes. The selective reduction of transcription not required for normal growth, but required for stress responses, likely affords additional resources for the production of exogenic proteins. In *yjl175w∆* cells, total resources for protein production were unchanged compared with those in WT cells; however, the allocation of these resources was found to be altered.

Swi3 is a subunit of the SWI/SNF chromatin remodeling complex that regulates transcription by remodeling chromosomes. The deletion of this subunit thereby causes significant changes in the transcriptional profiles of many genes^16^ (Fig. 2). The N-terminal deletion of Swi3 created by *yjl175w∆* leaves three important domains associated with Swi3 function, and transcriptional change via *yjl175w∆* is less pronounced than that via *swi3∆* (Fig. 2C). Interestingly, the N-terminal deletion does not result in milder transcriptional change but instead yields a different transcriptional profile from that of *swi3∆*, such as opposing directional changes observed in the transcripts of ribosomal proteins (Fig. S4). This finding indicates that the N-terminus of Swi3 might influence a specific set of genes.

A hallmark of cancer cells is an increase in chromosome number that triggers a massive overproduction of proteins^10,11^. Cancer cells must thereby evolve to overcome the protein burden effect. At least 20% of all human cancers contain mutations in the SWI/SNF complex including Swi3 homolog BAF155/SMARCC1 and BAF170/SMARCC2^18,30,31^. Loss of expression or the C-terminal truncation of BAF155 is associated with the proliferation of human cancer cell lines^32^. While mutations of Swi3 homologs found in cancer cells are not entirely same as the N-terminal deletion of Swi3 described here in yeast, it might create a similar situation to that of evolved cancer cells, that is, mutations in the SWI/SNF complex associated with an extensive transcriptional reduction and mitigation of the protein burden effect, facilitating rapid growth.

## Materials and Methods

### Strains used in this study

Strains used in this study are listed in Table 3. The *yjl175w∆* deletion cassette was generated by PCR using the genome of *yjl175w∆* strain in the yeast knockout collection (Horizon) as a template with the primers 5′-CGGCCGCTCTAGAACTAGTGGATCCGATGGAATTTCTTTGTAAACGCA-3′ and 5′-ATTGGGTACCGGGCCCCCCCTCGAGGCCCAAAAACGTATCTCTGCTTA. The cassette was cloned into the pRS413 vector via gap-repair cloning method in yeast. The C-terminal coding region of truncated *SWI3* in the plasmid was fused in-frame to the TAP tag generated by PCR using a TAP collection strain as a template (Dharmacon) and gap-repair cloning in yeast. PCR reactions for *yjl175w∆*-TAP cassette were performed with the primers 5′-GATGGAATTTCTTTGTAAACGCATT-3′ and 5′-GCCCAAAAACGTATCTCTGCTTAAA-3′. BY4741 was transformed with the *yjl175w∆*-TAP cassette by homologous recombination and selected on yeast extract/peptone/dextrose (YPD) plates containing 200 µg/mL G418. Insertion of the cassette was verified by genomic PCR with primers external to the integration locus, 5′-GACCGTTCCCAGTTAAGGTCGA-3′ and 5′-CGCTGCCAATGCTGAAGTATGT-3′.

**Table 3:** Strains used in this study.

Strain	Genotype	Reference
BY4741	*MATa his3Δ1 leu2Δ0 met15Δ0 ura3Δ0*	Brachman *et al*.^39^
ygl024w∆	*MATa ygl024wΔ::KanMX his3Δ1 leu2Δ0 met15Δ0 ura3Δ0*	Winzeler *et al*.^40^
yjl175w∆	*MATa yjl175wΔ::KanMX his3Δ1 leu2Δ0 met15Δ0 ura3Δ0*	Winzeler *et al*.^40^
swi3∆	*MATa yjl176cΔ::KanMX his3Δ1 leu2Δ0 met15Δ0 ura3Δ0*	Winzeler *et al*.^40^
ykl053w∆	*MATa ykl053wΔ::KanMX his3Δ1 leu2Δ0 met15Δ0 ura3Δ0*	Winzeler *et al*.^40^
SWI3-TAP	*MATa SWI3*-*TAP::HIS3MX6 leu2Δ0 met15Δ0 ura3Δ0*	Ghaemmaghami *et al*.^41^
yjl175w∆-TAP	*MATa yj175wΔ-TAP::KanMX his3Δ1 leu2Δ0 met15Δ0 ura3Δ0*	This study

### Growth conditions and yeast transformation

Yeast culture and transformation were performed as previously described^33^. YPD medium was used for yeast culture. Synthetic complete (SC) medium lacking uracil (–Ura) or leucine and uracil (−Leu/Ura) was used to culture yeast cells harboring plasmids. Strains used in this study are listed in Table 1. BY4741 was used as WT control. GFP is overproduced under control of the *TDH3* promoter on the 2-µm plasmid pTOW40836^34^. pTOW40836 was used as empty vector control.

### RNA-seq analysis

Sample preparation for RNA-seq was performed as described in Takasaki *et al*.^35^. BY4741 and *yjl175w∆* were grown in SC − Ura at 30 °C and sampled during the logarithmic growth phase. Cells were collected by centrifugation at 10,000 *g* for 10 min. Total RNA from 0.5 g of collected yeast cell samples was extracted using a FastRNA Pro Soil-Direct Kit (Qbiogene) according to the manufacturer’s instructions. Samples were treated with DNase to remove genomic DNA from total RNA with Recombinant DNaseI (RNase-free) (TaKaRa). After ethanol precipitation, the purified total RNA was stored at −80 °C until use in subsequent experiments. RNA purity and concentrations were estimated with a NanoDrop spectrophotometer (Thermo Fisher Scientific). RNA quality and quantity were estimated with a Bioanalyzer (Agilent, Tokyo, Japan). cDNA from purified mRNA was synthesized using a cDNA Synthesis Kit (TaKaRa) according to defined protocols. Briefly, first, poly(A) RNA was reverse-transcribed with an oligo (dT)-T7 primer containing a T7 promoter sequence; this was used to synthesize double-stranded cDNA. cDNA templates were then transcribed *in vitro* with T7 RNA polymerase (TaKaRa), yielding large amounts of antisense RNA (aRNA). Finally, aRNA was further reverse-transcribed to cDNA with a biotinated oligo (dT) primer for next generation sequencing. Sequencing of the synthesized cDNA was performed by paired-end sequencing on an Illumina Hiseq2000 sequencing system provided by the Hokkaido System Science Co., Ltd. We analyzed three biological replicates for each strain.

### Transcriptome data analysis

RNA-seq data of Swi3 knockout and WT strains (GEO, ID: 302174480, 302174481, 30174486, and 302174487)^16^ were downloaded from the SRA database. Our RNA-seq data of deletion of *YJL175W* and WT are available from the SRA database (SRA, ID: SRR10848971 and SRR10848972). All RNA-seq data, including ours and Dutta’s, were aligned to the Ensembl R64-1-1 genome using HISAT2 version 2.1.0 with gene annotations from Ensembl R64-1-1. HISAT2 options were *-p 8 –dta*. Ensembl R64-1-1 was obtained from iGenomes by Illumina, Inc. Visualizations of mapped fragments were conducted with an Integrative Genome Viewer (2.4.9). Assembly and estimation of transcript abundances were performed with HTSeq version 0.11.1. Downstream analysis was conducted using Python (3.6.8). Transcripts per kilobase millions (TPMs) were calculated according to a previously described method^36^. Calculated TPM values are provided in Tables S2 and S3.

### TAP-tag western blot

Detection of TAP-tag protein by Western blot was performed as described in Ishikawa *et al*.^37^. Yeast strains were aerobically cultured at 30 °C in 2 mL of YPD medium. Optical density at 660 nm (OD_660_) was measured, and units of 1 OD_660_ were harvested during the log phase. Cells were treated with 1 mL of 0.2 N NaOH for 5 min at room temperature and then suspended in 2× NuPAGE LDS sample buffer (Invitrogen) and heated to 70 °C for 10 min. Protein lysate in the supernatant was labeled with EzLabel FluoroNeo (ATTO) and subjected to polyacrylamide gel electrophoresis with lithium dodecyl sulfate (SDS-PAGE), followed by Western blot with PAP (Sigma-Aldrich) (1:2000) and peroxidase-conjugated secondary antibody (Nichirei Biosciences) (1:1000). We used a NuPAGE 4–12% Bis-Tris Gel (Invitrogen) for SDS-PAGE and an iBlot Transfer Stack PVDF membrane (Invitrogen) for Western blot. Chemiluminescence was induced by SuperSignal West Femto Maximum Sensitivity Substrate (Thermo Fisher Scientific) and detected on a LAS-4000 image analyzer (Fujifilm) using ImageQuant LAS 4000 (GE Healthcare).

### Polysome profiling

Frozen yeast cells were mixed with frozen droplets of 600 µL lysis buffer (20 mM Tris–HCl (pH 7.5), 150 mM NaCl, 5 mM MgCl_2_, 1 mM dithiothreitol, 100 µg/mL cycloheximide, and 1% Triton X-100) and lysed with Multi-beads Shocker (Yasui Kikai). Lysates were treated with 25 U of TURBO DNase (Thermo Fisher Scientific) and cleared by centrifugation at 20,000 *g* for 10 min at 4 °C. RNA concentration in the lysate was measured with a Qubit RNA BR Assay Kit (Thermo Fisher Scientific). Sucrose gradients (10–50% sucrose in lysis buffer without Triton X-100) were prepared in 14 × 95 mm open-top Polyclear centrifuge tubes (SETON) using a Gradient Station (BioComp). Lysates containing 20 µg RNA were loaded on top of the sucrose gradients and centrifuged at 35,300 rpm for 2.5 h at 4 °C using a rotor P40ST (Hitachi Koki). After ultra-centrifugation, the absorbance at 254 nm was measured continuously on a Bio-mini UV monitor (ATTO).

### Statistical analysis

Where appropriate, values are expressed as mean ± SD. Statistical analyses of RNA-seq date from three biological replicates were conducted using the Benjamini–Hochberg method, and a false discovery rate <0.05 was considered statically significant^38^. Statistical analyses with more than two groups were performed by Bonferroni correction. An adjusted *p*-value of <0.05 was considered statistically significant. All data are representative of multiple repeated experiments.

## Supplementary information

Supplementary information.

Supplementary information 2.

Supplementary information 3.

Supplementary information 4.

## References

[1] Sopko R (2006). Mapping pathways and phenotypes by systematic gene overexpression. Mol. Cell.

[2] Makanae K, Kintaka R, Makino T, Kitano H, Moriya H (2013). Identification of dosage-sensitive genes in Saccharomyces cerevisiae using the genetic tug-of-war method. Genome Res..

[3] Moriya H (2015). Quantitative nature of overexpression experiments. Mol. Biol. Cell.

[4] Kafri M, Metzl-Raz E, Jona G, Barkai N (2016). The Cost of Protein Production. Cell Rep.

[5] Snoep JL, Yomano LP, Westerhoff HV, Ingram LO (1995). Protein burden in Zymomonas mobilis: Negative flux and growth control due to overproduction of glycolytic enzymes. Microbiology.

[6] Eguchi Y (2018). Estimating the protein burden limit of yeast cells by measuring the expression limits of glycolytic proteins. Elife.

[7] Kurland CG, Dong H (1996). Bacterial growth inhibition by overproduction of protein. Mol. Microbiol..

[8] Farkas Z (2018). Hsp70-associated chaperones have a critical role in buffering protein production costs. Elife.

[9] Frumkin I (2017). Gene Architectures that Minimize Cost of Gene Expression. Mol. Cell.

[10] Tang YC, Amon A (2013). Gene copy-number alterations: A cost-benefit analysis. Cell.

[11] Ben-David, U. & Amon, A. Context is everything: aneuploidy in cancer. *Nat. Rev. Genet* **21**, 44–62 (2020). 10.1038/s41576-019-0171-x 31548659

[12] Kintaka R, Makanae K, Moriya H (2016). Cellular growth defects triggered by an overload of protein localization processes. Sci. Rep.

[13] Kintaka, R. *et al*. Genetic Profiling of Protein Burden and Nuclear Export Overload. *bioRxiv* 2020.02.25.962068 (2020). 10.7554/eLife.54080 PMC7673788 33146608

[14] Kassabov SR, Zhang B, Persinger J, Bartholomew B (2003). SWI/SNF unwraps, slides, and rewraps the nucleosome. Mol. Cell.

[15] Phelan ML, Sif S, Narlikar GJ, Kingston RE (1999). Reconstitution of a core chromatin remodeling complex from SWI/SNF subunits. Mol. Cell.

[16] Dutta A (2017). Composition and Function of Mutant Swi/Snf Complexes. Cell Rep.

[17] Helming KC, Wang X, Roberts CWM (2014). Vulnerabilities of mutant SWI/SNF complexes in cancer. Cancer Cell.

[18] Kadoch C (2013). Proteomic and bioinformatic analysis of mammalian SWI/SNF complexes identifies extensive roles in human malignancy. Nat. Genet..

[19] Wilson BG, Roberts CWM (2011). SWI/SNF nucleosome remodellers and cancer. Nat. Rev. Cancer.

[20] Baryshnikova, A. *et al*. *Synthetic genetic array (SGA) analysis in Saccharomyces cerevisiae and schizosaccharomyces pombe*. *Methods in Enzymology* **470**, (Elsevier Inc., 2010). 10.1016/S0076-6879(10)70007-0 20946810

[21] Peterson CL, Herskowitz I (1992). Characterization of the yeast SWI1, SWI2, and SWI3 genes, which encode a global activator of transcription. Cell.

[22] Boyer LA (2002). Essential role for the SANT domain in the functioning of multiple chromatin remodeling enzymes. Mol. Cell.

[23] Da G (2006). Structure and function of the SWIRM domain, a conserved protein module found in chromatin regulatory complexes. Proc. Natl. Acad. Sci. USA.

[24] Kuras L, Cherest H, Surdin-Kerjan Y, Thomas D (1996). A heteromeric complex containing the centromere binding factor 1 and two basic leucine zipper factors, Met4 and Met28, mediates the transcription activation of yeast sulfur metabolism. EMBO J..

[25] Blaiseau PL, Isnard AD, Surdin-Kerjan Y, Thomas D (1997). Met31p and Met32p, two related zinc finger proteins, are involved in transcriptional regulation of yeast sulfur amino acid metabolism. Mol. Cell. Biol..

[26] Servant G (2012). Tye7 regulates yeast Ty1 retrotransposon sense and antisense transcription in response to adenylic nucleotides stress. Nucleic Acids Res..

[27] Laloux I, Dubois E, Dewerchin M, Jacobs E (1990). TEC1, a gene involved in the activation of Ty1 and Ty1-mediated gene expression in Saccharomyces cerevisiae: cloning and molecular analysis. Mol. Cell. Biol..

[28] Costa EA, Subramanian K, Nunnari J, Weissman JS (2018). Defining the physiological role of SRP in protein-targeting efficiency and specificity. Science (80−).

[29] Giaever, G. *et al*. Functional profiling of the Saccharomyces cerevisiae genome. *Nature* 387–391 (2002). 10.1038/nature00935 12140549

[30] Biegel JA, Busse TM, Weissman BE (2014). SWI/SNF chromatin remodeling complexes and cancer. Am. J. Med. Genet. Part C Semin. Med. Genet.

[31] Shain, A. H. & Pollack, J. R. The Spectrum of SWI/SNF Mutations, Ubiquitous in Human Cancers. *PLoS One* **8** (2013). 10.1371/journal.pone.0055119 PMC3552954 23355908

[32] del Bove J (2011). Identification of a core member of the SWI/SNF complex, BAF155/SMARCC1, as a human tumor suppressor gene. Epigenetics.

[33] Amberg, D. C. & Strathern, J. N. *Methods in yeast genetics: a Cold Spring Harbor Laboratory course manual*. (CSHL press, 2005).

[34] Moriya H, Makanae K, Watanabe K, Chino A, Shimizu-Yoshida Y (2012). Robustness analysis of cellular systems using the genetic tug-of-war method. Mol. Biosyst..

[35] Takasaki, K. *et al*. Discovery of Glycoside Hydrolase Enzymes in an Avicel-Adapted Forest Soil Fungal Community by a Metatranscriptomic Approach. *Plos One* **8** (2013). 10.1371/journal.pone.0055485 PMC3564753 23393585

[36] Li B, Ruotti V, Stewart RM, Thomson JA, Dewey CN (2009). RNA-Seq gene expression estimation with read mapping uncertainty. Bioinformatics.

[37] Ishikawa K, Makanae K, Iwasaki S, Ingolia NT, Moriya H (2017). Post-Translational Dosage Compensation Buffers Genetic Perturbations to Stoichiometry of Protein Complexes. PLoS Genet..

[38] Benjamini, Y. & Hochberg, Y. Controlling the False Discovery Rate: A Practical and Powerful Approach to Multiple Testing Yoav Benjamini; Yosef Hochberg Journal of the Royal Statistical Society. Series B (Methodological), Vol. 57, No. 1. (1995), pp. *J. R. Stat. Soc*. **57**, 289–300 (1995).

[39] Brachman CB (1998). Designer Deletion Strains derived from Saccharomyces cerevisiae S288C: a Useful set of Strains and Plasmids for PCR-mediated Gene Disruption and Other Applications. Yeast.

[40] Winzeler EA (1999). Functional characterization of the S. cerevisiae genome by gene deletion and parallel analysis. Science (80–).

[41] Ghaemmaghami, S. *et al*. Global analysis of protein expression in yeast. *Nature* **425**, 737–41 (2003). 10.1038/nature02046 14562106

