# Supplementary material for: N-terminal deletion of Swi3 created by the deletion of a dubious ORF YJL175W mitigates protein burden effect in S. cerevisiae
Source: Sci Rep. 2020 Jun 11;10:9500. doi: 10.1038/s41598-020-66307-z (PMC7289859; doi:10.1038/s41598-020-66307-z)
Supplement: Supplementary file 4 — Supplementary information 4. [file 41598_2020_66307_MOESM4_ESM.pdf]

Supplementary Information (Figure S1-S5) for

N-terminal deletion of Swi3 created by the deletion of a dubious ORF *YJL175W* mitigates protein burden effect in *S. cerevisiae*

Nozomu Saeki[1], Yuichi Eguchi[2], Reiko Kintaka[3], Koji Makanae[1], Yuichi Shichino[4], Shintaro Iwasaki[4,5], Manabu Kanno[6], Nobutada Kimura[6], Hisao Moriya[1, 7, *]

[1]Graduate School of Environmental and Life Science, Okayama University, Okayama Japan

[2]Center for Mechanisms of Evolution, School of Life Sciences, Arizona State University, Arizona, USA

[3]Donnelly Center for Cellular and Biomolecular Research, Department of Medical Genetics, University of Toronto, Toronto, Canada

[4]RNA Systems Biochemistry Laboratory, RIKEN Cluster for Pioneering Research, Saitama, Japan

[5]Department of Computational Biology and Medical Sciences, Graduate School of Frontier Sciences, The University of Tokyo, Chiba, Japan

[6]Bioproduction Research Institute, National Institute of Advanced Industrial Science and Technology, Ibaraki, Japan

[7]Research Core for Interdisciplinary Sciences, Okayama University, Okayama, Japan

* Correspondence to: hisaom@cc.okayama-u.ac.jp

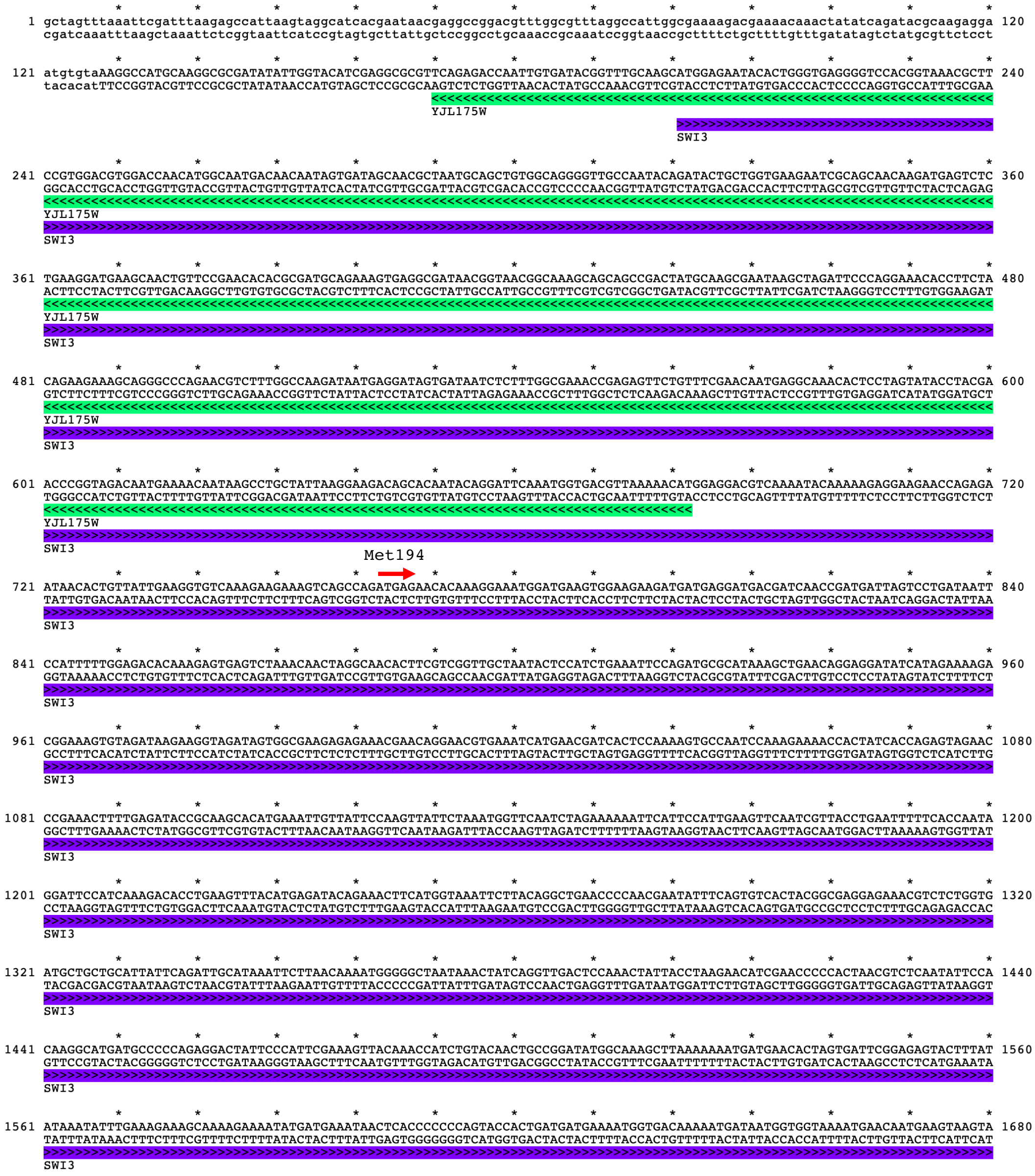

Figure S1. The DNA sequence of *YJL175W-SWI3* locus of the wild type strain from 200 bp upstream of the SWI3 start codon. Corresponding positions of *YJL175W* and *SWI3* (N-terminal region) are colored. The predicted start codon of *swi3ΔN* is shown as Met194.

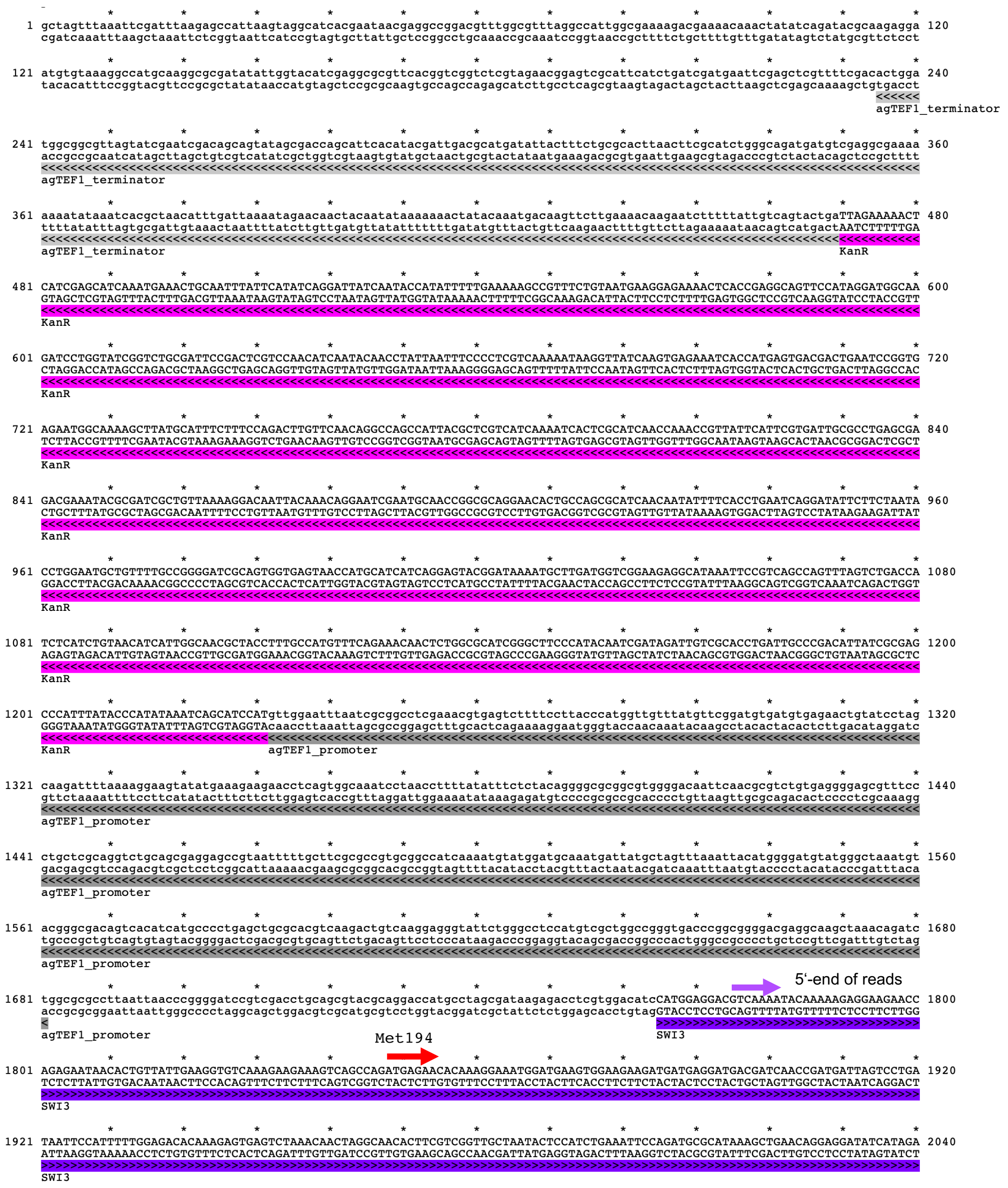

Figure S2. The DNA sequence of *YJL175W-SWI3* locus of the *YJL175W* deletion strain from 200 bp upstream of the *SWI3* start codon. Corresponding positions of the *KanMX4* cassette (*KanR* and *agTEF1* promoter and terminator) used for the deletion and *SWI3* (N-terminal region) are colored. The predicted start codon of *swi3ΔN* is shown as Met194.

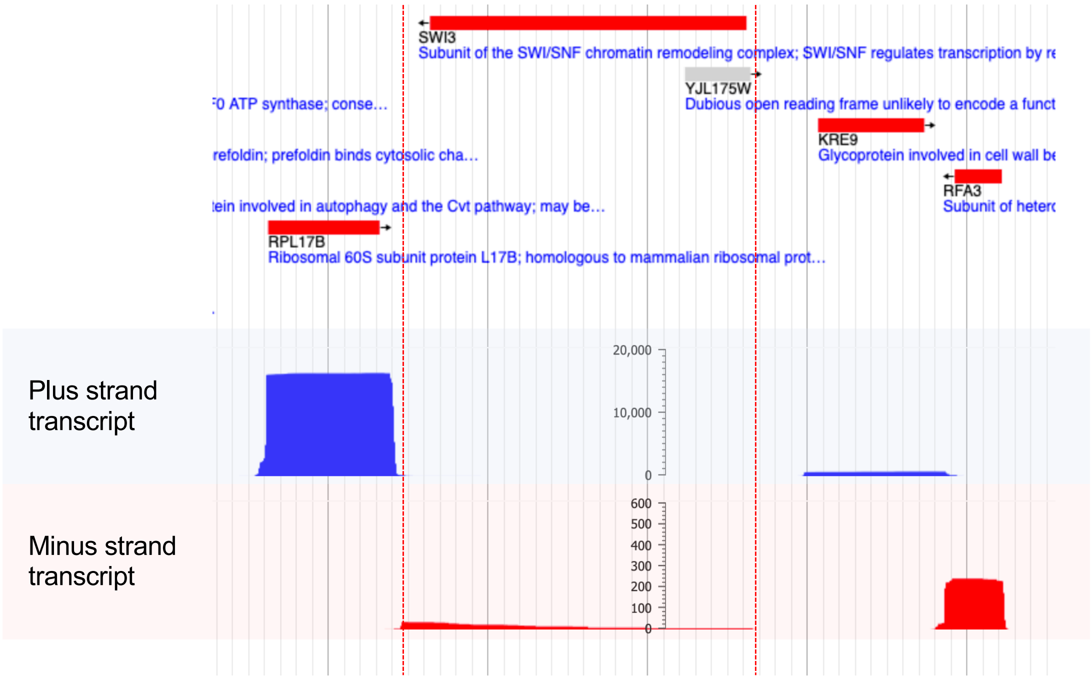

Figure S3. Transcripts detected in the *YJL175W-SWI3* genomic region. While transcripts corresponding to *SWI3* are detected (shown between red-dotted lines), those to *YJL175W* was not. The screenshot was taken from https://browse.yeastgenome.org/ (created by JBrowse ver. 1.12.1). The visualized transcripts data was from Pelechano et al., 2013[1].

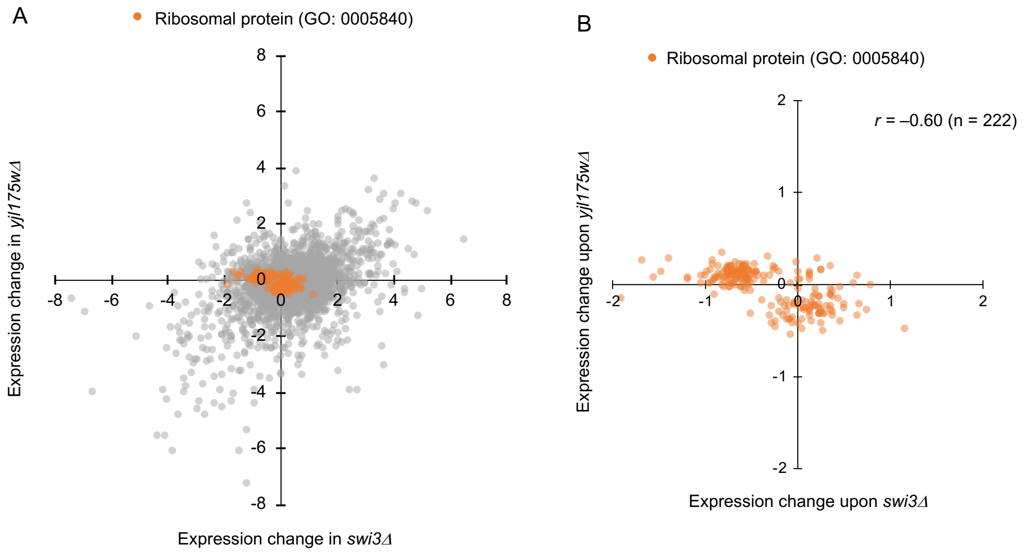

Figure S4. Expression changes in *swi3*Δ and *yjl175w*Δ. (**A**) Expression changes of transcripts encoding ribosome proteins in *swi3*Δ and *yjl175w*Δ plotted on all other transcripts shown as gray spots. (**B**) Expression changes of transcripts of ribosomal proteins in *swi3*Δ and *yjl175w*Δ.

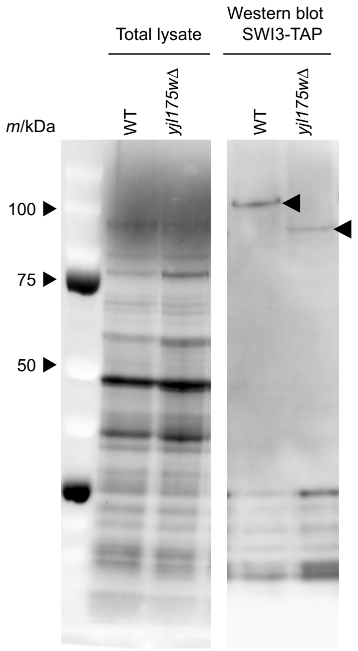

Figure S5. The full-length gel and blot images used in Figure 2B.

Reference

1. Pelechano, V., Wei, W. & Steinmetz, L. M. Extensive transcriptional heterogeneity revealed by isoform profiling. *Nature* **497**, 127–131 (2013).
